# Differential Treatment Responses of Maltreated and Neglected Children and Adolescents Following an Evidence-based Multisystemic Intervention

**DOI:** 10.1007/s10802-024-01248-z

**Published:** 2024-10-14

**Authors:** Corinna Buderer, Tom Kirsch, Tania Pérez, Cynthia Cupit Swenson, Marc Schmid

**Affiliations:** 1 Clinic of Child and Adolescent Psychiatry and Psychotherapy, Psychiatric Services Aargau AG, Windisch, Switzerland; 2 Department of Child and Adolescent Psychiatry, Psychiatric University Clinics Basel, University of Basel, Basel, Switzerland; 3 Division of Global and Community Health, Medical University of South Carolina, Charleston, USA

**Keywords:** Multisystemic Therapy for Child Abuse and Neglect (MST-CAN), Child behavior checklist, Treatment outcome, Psychotherapy, Subgroups, Person-centered approach

## Abstract

Limited studies have investigated differential treatment responses to family-based treatment programs and subgroup trajectories in youth in a high-risk context. This study pioneered an examination of Multisystemic Therapy for Child Abuse and Neglect (MST-CAN) and built on prior research that identified subgroups with different psychopathologies. Participants included 208 parent–child dyads enrolled in the MST-CAN evaluation in Switzerland. Parents reported their children’s (*M*_age_ = 10.27 years, *SD*_age_ = 3.5, 44.2% girls, 55.8% boys, 98.6% White) emotional and behavioral problems. Longitudinal data were examined to analyze the differential changes within the pre- and post-treatment (T1 and T2) subgroups. The T1 cluster and T2 cluster were cross-tabulated to examine changes in the symptom class over time. Overall, the treatment proved to be highly beneficial. Subgroup analyses revealed that four out of the five subgroups (80%) showed positive changes in at least two outcome measures. The treatment was most successful for children with externalizing symptoms. Children with multiple symptoms also showed improvements across different symptoms. Regarding specific symptoms, children with anxious-avoidant symptoms benefited from the treatment. Additionally, the treatment was beneficial for children with normative emotions and behavior. Meanwhile, the treatment did not have any significant effects for children with internalizing symptoms. Notably, child neglect was reduced in three (60%) subgroups. The symptom class remained stable across time for children with externalizing and multiple symptoms. Ultimately, MST-CAN reduced emotional and behavioral problems and child neglect in most families. Understanding children’s differential treatment responses to complex treatment programs is essential to adequately address different needs.

**Supplementary Information:**

The online version contains supplementary material available at 10.1007/s10802-024-01248-z.

## Introduction

Children and adolescents experiencing maltreatment in their families are at an increased risk of developing various mental health problems with broad comorbidities (Bürgin et al., 2023; Schmid et al., 2013). Substantial evidence shows associations between child maltreatment and externalizing and internalizing problems (Hunt et al., 2017; Jaffee, 2017). The long-term consequences of child maltreatment have been linked to various mental health disorders, such as depressive and anxiety disorders, post-traumatic stress disorder, drug use, and suicide attempts (Jaffee, 2017; Mehta et al., 2021; Norman et al., 2012). Besides mental health, child maltreatment can also have a substantial impact on other domains, such as physical health (Anda et al., 2006), delinquent behavior in adulthood (Baglivio et al., 2020), and difficulties related to professional development, finances (Copeland et al., 2018) social integration, and relationships (Lo et al.,  2019); therefore, child maltreatment can impair the entire life course (Danese & Baldwin, 2017; Schmid et al., 2022). Complex treatment programs are needed to mitigate these effects.

Currently, research on how treatment programs address the varying symptomatology of high-risk children and adolescents is scarce. In particular, complex treatments must prove effective across various age groups, mental health issues, and family crises. These programs should target various psychopathologies in both children and adolescents, as well as parental factors, to improve parent–child interactions and reduce the risk of the (re-)occurrence of child abuse and neglect. For manual-based treatments, understanding the patients and families who would benefit is important.

Multisystemic Therapy for Child Abuse and Neglect (MST-CAN) (Swenson et al., 2010) is one example of this kind of treatment program and is designed for families that have experienced physical abuse and/or neglect (Bauch et al., 2022; Buderer et al., 2020; Hefti et al., 2020; Swenson et al., 2010). As a family intervention, MST-CAN is an adaptation of Standard Multisystemic Therapy (MST; Hengeler et al., 2009); in particular, MST-CAN focuses on the caregiver’s behavior, which triggers enrollment in the program. The program aims to reduce and prevent the (re-)occurrence of child abuse and neglect and avoid out-of-home placements for children. Furthermore, the program targets mental health difficulties of both children and parents. Like the MST Standard, the MST-CAN is based on Bronfenbrenner’s (1979) socioecological model. The MST-CAN combines evidence-based systemic and cognitive-behavioral interventions with case management within the family’s living environment. Swenson et al. (2010) demonstrated its superiority over an Enhanced Outpatient Treatment in a randomized controlled trial (RCT) on diverse outcome measures at the child and parental levels.

While studies on differential treatment outcomes in high-risk children and adolescents are increasingly important, they are rarely investigated (Nagin & Odgers, 2010). To date, meta-analyses have concentrated on the overall effectiveness of treatment programs for preventing the recurrence of child maltreatment (Euser et al., 2015; Gubbels et al., 2019; van der Put et al., 2018). However, heterogeneity across families and child mental health needs suggest that treatment outcomes may vary.

A more person-centered approach contrasts with therapists’ adherence to treatment. Meta-analyses have shown how challenging it is to adhere to treatment and, further, that adherence is not associated with symptom changes as treatment outcomes (Collyer et al., 2020; Webb et al., 2010). Nevertheless, three MST Standard studies have found strong associations between adherence to the MST process and treatment outcomes (Huey et al., 2000; Schoenwald et al., 2008), which extended to legal records years after treatment (Schoenwald et al., 2009).

### Differential Treatment Outcomes in the Context of MST

In the MST context, to our best knowledge, only four studies have examined differential treatment responses and change trajectories via different approaches. Halliday-Boykins et al. (2004) conducted an RCT that examined 156 adolescents who had experienced a suicidal crisis and subsequently received MST Standard. Based on the adolescents’ psychopathological symptoms, they identified five different developmental trajectories: high improvement, high unimprovement, borderline improvement, borderline unimprovement, and subclinical. They concluded that, contrary to the general assumption, youths with severe psychopathology were at risk of maintaining their symptoms at a high level and benefited less from treatment (Halliday-Boykins et al., 2004). Mertens et al. (2017) investigated various treatment outcomes related to externalizing problem behaviors in 147 adolescents treated with MST Standard in the Netherlands, using data from an RCT on the effectiveness of MST Standard. They identified six subgroups, each with different developmental trajectories. Of these, four subgroups benefited from treatment, one showed no changes, and one deteriorated. The authors emphasized the need for individualized treatment based on these results. Keles et al. (2021) found in a naturalistic treatment outcome study heterogeneous trajectories of treatment responses among 1674 adolescents with serious and persistent antisocial behavior treated with MST Standard in Norway. Buderer et al.’s (2024) naturalistic treatment outcome study identified five distinct symptom groups in children and adolescents from 194 families referred and treated with MST-CAN in Switzerland: (a) children with anxious-avoidant symptoms, (b) children with multiple symptoms, (c) children with predominantly externalizing symptoms, (d) children without psychopathological findings, and (e) children with mainly internalizing symptoms. This study provided preliminary evidence that children and families benefited equally with respect to the overarching goals of MST-CAN (the child still lived at home and went to school, there were no new charges against the parents and no new Child Protections Services (CPS) reports). However, differential treatment responses of emotional and behavioral problems within the subgroups and subgroup changes were not examined.

### Differential Treatment Outcomes and Trajectories of Emotional and Behavioral Problems in Children and Adolescents Undergoing Interventions

Based on a meta-analysis and individual studies that examined differential treatment responses and trajectories in children and adolescents, children and adolescents with different symptom classes differently benefited from treatment and exhibited distinct trajectories (Keles et al., 2021; Pasalich et al., 2022; Weisz et al., 2017). According to the meta-analysis, children and adolescents with anxiety benefited the most from the psychological treatment, those with depression benefitted the least, those with externalizing symptoms benefited moderately, and those with multiple problems did not significantly benefit (Weisz et al., 2017). However, these findings were not specific to high-risk children.

Outside the MST context, Pasalich et al. (2022) investigated the differential treatment responses of adolescents with severe behavioral and mental health problems. In total, 487 youths and 682 parents were enrolled in an attachment-based and trauma informed parent program. Those with severe externalizing behavior benefited the most, whereas those with comorbid externalizing and internalizing problem behaviors showed only a partial or moderate response to the treatment. Most youths with moderate or low levels of externalizing and internalizing problem behaviors at baseline gradually improved. These studies suggested that different groups of adolescents benefited from family-based treatments in various ways. However, these studies did not provide information on how symptoms changed. To the best of our knowledge, only one study examined symptom changes in families within a high-risk context. Zhang and Slesnick (2018) identified four classes of internalizing and externalizing behavior (internalizing only, externalizing only, comorbid, and normative) in children with substance-misusing parents who received family systems therapy. Follow-up results showed that, after 18 months, children in the externalizing class were more likely to be in the normative class and those in the comorbid class more likely to be in the internalizing class. This study indicated changes in symptom classes for certain groups; however, generalizable statements could not be made.

In sum, three studies found variability in various outcome measures for the MST Standard. For MST-CAN, only one study provided initial indications for subgroups of children with different mental health needs. Another study outside the MST context provides further evidence for heterogeneous treatment outcomes within the framework of a trauma-informed, family-based intervention for children and adolescents. Only one study investigated the change in externalizing and internalizing symptoms in response to treatment in children and adolescents of a high-risk population. The results are not easily transferable as changes might vary in different settings, modalities, and problems (Warren et al., 2010). To the best of our knowledge, no previous study has examined differential treatment responses for child neglect, especially for child neglect in families with multiple, complex needs. This study is the first to examine this topic for emotional and behavioral problems as well as child neglect.

### This Study

This study aimed to investigate changes in emotional and behavioral problems and the severity of child neglect in subgroups of children and adolescents treated with MST-CAN, both cross-sectionally and longitudinally, via a combination of variable-and person-centered approaches. For the person-centered approach, we followed Bergman and Magnusson’s (1997) theoretical assumptions for cluster analyses. We assumed heterogeneity in emotional and behavioral problems among the children and adolescents referred to and treated with MST-CAN. We adopted the subgroups, including the labeling from a previous study by Buderer et al. (2024), reported above. In addition, we hypothesized that within these subgroups, there would be different treatment outcomes and changes in psychopathologies. Therefore, we investigated the following research questions using a two-fold approach:

1. Are there differential changes in emotional and behavioral problems (dependent variable) and the severity of child neglect (dependent variable) within subgroups, following prior research?
2. Do subgroups (symptom cluster) change between two assessment points?
  - Which subgroups (symptom cluster) can we identify at the end of the treatment?
  - Do similar or different subgroups (symptom cluster) emerge at each assessment point? (structural changes or stability)
3. Do children and adolescents who belong to a specific subgroup (symptom cluster) before treatment tend to belong to a similar or different subgroup (symptom cluster) after treatment? (individual changes or stability)
4. If subgroups are identified after treatment, do the subgroups differ in terms of their characteristics?

## Methods

### Participants and Procedure

Participants were 208 parent–child dyads from families referred to MST-CAN in Switzerland between 2011–2023 by CPS. Families were referred based on a report of physical abuse and/or neglect in the preceding 180 days, as documented by a social worker. The inclusion criteria for the intervention program were cases with a target child aged between 6 and 17 years, who was not acutely suicidal, homicidal, psychotic, or diagnosed with autism spectrum disorder level 2 or 3 (DSM; American Psychiatric Association, 2013); and who was either living with their family or in foster care with the prospect of being rapidly reunited with their family. Cases with active sexual abuse, severe domestic violence, and parental psychosis were excluded.

Target children and parents were invited to participate. Of the 313 families approached, 214 parents provided informed consent before study participation. Based on intervention dropouts, owing to a lack of engagement (n = 15, 7.0%), out-of-home placements (n = 5, 2.3%), administrative withdrawals (n = 4, 1.9%), a lack of funding (n = 3, 1.4%), and family relocation (n = 2, 0.9%) (Appendix A), we excluded 26 parent–child dyads (12.1%). We further excluded six parent–child dyads with missing values for the CBCL scores. Hence, the final sample consisted of 208 parent–child dyads. Families who dropped out did not differ significantly from those who completed the intervention program in terms of demographic measures (i.e., age, sex assigned at birth, and migration background), severity of initial child neglect, and children’s emotional and behavioral problems. These families were treated between 24 therapists. The number of treated families per therapist varied from one family (0.5%) to 32 families (15.4%).

The demographic variables for the overall sample are detailed in Table 1.

**Table 1:** Descriptive characteristics of the sample

Characteristics	*n* (%)	*M (SD)*
Child age		10.27 (3.5)
Female	92 (44.2%)	
Male	116 (55.8%)	
White (Swiss, other European Countries, America, North Africa)	205 (98.6%)	
Asian	3 (1.4%)	
Migration background (1st or 2nd generation immigrants)	76 (36.5%)	
Single parenthood	105 (50.5%)	
Parent unemployed	85 (40.9%)	
Parent without school graduation	13 (6.3%)	
Multiple children in the household	90 (43.3%)	
Child neglect as the reason for referral	71 (34.1%)	
Child abuse as the reason for referral	71 (36.6%)	
Parents noticeably overwhelmed, maltreated detected during treatment	56 (27.3%)	
Prior removal of custody	13 (6.3%)	
Child’s emotional and behavioral problem is clinically significant	124 (59.6%)	

*CPS* Child protection service, *M* Mean, *SD* Standard deviation

### Intervention

MST-CAN was led by a single therapist who was integrated into a team of three to four therapists; caseloads comprised three to four families per therapist. Families received two to three weekly treatment sessions within their homes with a duration of six to nine months. Pharmacotherapy was provided by a child and adolescent psychiatrist when deemed necessary. To address crises occurring outside regular working hours, a 24/7 on-call service was available. Additionally, weekly consultations with an MST-CAN expert were held to ensure adherence to the treatment model. The team received support from a family resource specialist and was overseen by a team supervisor, with both individual and group supervision sessions conducted weekly. All therapists completed extensive training, including a five-day MST training (Henggeler et al., 2009), a four-day MST-CAN training, and a four-day trauma therapy training for adults and children. Booster trainings took place every three months for all therapists and were carried out by the MST-CAN expert to ensure the ongoing quality of the intervention program. Treatment fidelity was ensured using the Adherence Scale-Revised for Child Abuse and Neglect (TAM – CAN – R; Swenson, 2010). Further information and results were reported in a previous study by Buderer et al. (2024).

For a further comprehensive understanding of the program and its nine core treatment principles, detailed descriptions can be found in Swenson et al. (2010), Swenson and Schaeffer (2018), and the MST-CAN manual (Swenson et al., 2011).

### Data Collection

This study was approved by local ethics committees (Ethikkommission Ostschweiz, Ethikkommission Nordwest- und Zentralschweiz). Data were collected from July 2011 to December 2018 in Thurgau and from November 2014 to November 2023 in Basel. Both oral and written informed consent was obtained from under-aged participants and legal guardians before study participation. With the help of a research assistant, the participating parents and children completed various questionnaires at the beginning and end of the treatment. Severity of child neglect was assessed externally via an interview with the case worker of the referring CPS at the beginning and end of the MST-CAN. The case worker reported the case characteristics and type and severity of maltreatment.

### Measures

#### Childhood Emotional and Behavioral Problems

Children’s emotional and behavioral problems were assessed via the Child Behavior Checklist (Achenbach, 1991; CBCL/4–18; Workgroup German Version of the Child Behavior Checklist, 1998) that comprised 113 items. Parents were asked if they had observed a specific behavior in their child and reported their answers on a 3-point Likert scale that ranged from “not true” (0), “somewhat true” (1), or “always or often true” (2). Items were summarized into eight subscales (social withdrawal, somatic complaints, anxiety/depression, social, thought, attention problems, and delinquent and aggressive behavior), two broadband scales (internalizing and externalizing), and a total score. For clinical purposes, the raw scores were converted into *T*-scores. For the three scales of internalizing, externalizing, and total problems, scores between 60–63 and > 63 were considered within the borderline clinical range and clinical range, respectively. In this sample, the internal consistency for the total score was excellent, with Cronbach’s *α* = 0.95.

#### Child Neglect

The Ontario Child Neglect Index (CNI; Trocmé, 1996) was used to measure the severity of neglect. The CNI comprised six items that reflected different forms of neglect (i.e., supervision, nutrition, clothing and hygiene, physical health, mental health, and development/educational care). Items were rated on a 4- to 5-level scale (i.e., “adequate,” “inconsistent,” “inadequate,” and “seriously inadequate”) by trained child welfare professionals. To calculate a total score that reflected the severity of neglect, the scale scores were summed and an age score ranging from 0 (13–16 years) to 20 (0–2 years) was added to the highest score among the six scales. The underlying model assumed that the six neglect items represented different forms of neglect rather than different components that could be added together. The total score ranged from 0–80 points, and higher scores indicated more severe levels of child neglect. This study used an unpublished German version of the CNI (Pérez et al., 2017), which was approved by Trocmé based on a back-translation into English.

### Data Analysis

Descriptive statistics and frequency distributions were calculated for demographic characteristics. In a preliminary analysis, we conducted paired-sample *t*-tests for the CBCL total scale and two broadband scales (internalizing problems and externalizing problems) and the CNI to analyze changes in the means between T1 (pre-treatment) and T2 (post-treatment). To answer our first research question, we repeated these analyses for each subgroup, which included the eight CBCL subscales. We applied the Bonferroni correction to obtain *p*-values. Cohen’s *d* was used as the effect size; an effect size of 0.20 was considered small, 0.50 medium, and 0.80 large (Cohen, 2013).

To answer the second and third research questions, we followed the Linking of Clusters after removal of a Residue (LICUR) method (Bergman et al., 2003), which is a pattern-analytical procedure, forming clusters at each measurement point to determine the individual development between the clusters of the different measurement points (Daukantaitė et al., 2019; Schmid et al., 2021; Viborg et al., 2018). This method comprises three steps. First, for each measurement point, the multivariate outliers are identified by means of a residue procedure and removed from further analyses to prevent the distortion of later cluster analyses. Second, Ward’s hierarchical clustering method (Ward, 1963) and Squared Euclidean distances are performed for each measurement point. Optimal cluster solutions are chosen based on the content aspects and statistical criteria recommended by Bergman et al. (2003). In our cluster analyses, the operation factors were the eight CBCL subscales.

Third, the subgroup trajectories between the measurement points are investigated regarding structural and individual stability/change. Distances of the cluster centroids at T1 and T2 were analyzed and compared by calculating the squared Euclidean distances. Small and larger distances referred to high similarity and dissimilarity, which indicated structural stability and structural change, respectively. To analyze individual stability/change, the two cluster solutions were cross-tabulated and the number of transitions expected with those actually observed were compared. Significance was assessed via Fisher’s exact test, with a hypergeometric distribution. To address mass significance, the Bonferroni correction was applied. Observed paths that were significantly higher than expected were referred to as developmental types. Observed paths that were lower than expected were referred to as developmental antitypes, which indicated that a transition was unlikely. The odds ratio (*OR*) was calculated to determine the extent to which the probability of a significant path increased (for developmental types; *OR* > 1.0) or decreased (for antitypes; *OR* < 1.0). Regarding the optimal sample size for cluster analysis, we relied on a recent recommendation by Dalmaijer et al., (2022), reasoning that sufficient statistical power for cluster analysis is assumed when the subgroups contain 20 to 30 cases.

Finally, we compared the demographic variables of the T2 clusters to determine their specific characteristics vis-a-vis. For continuous variables, we performed a one-way analysis of variance with post hoc comparisons via Tukey’s Honest Significant Differences. For nominal variables (sex assigned at birth, migration background, and single parenthood), we performed a chi-squared test.

IBM SPSS version 29 was used for data analysis. To analyze the individual development of the subgroups, we used the ExaCon module of the statistics package ROPstat 2.0 (Vargha et al., 2015).

## Results

### Preliminary Analyses

#### Changes in Emotional And Behavioral Problems and Child Neglect (Variable-Centered)

Paired sample *t*-tests were used to determine the average group changes over time. Significant changes were observed for emotional and behavioral problems and severity of child neglect (Tables 2 and 3).

**Table 2:** Descriptive analysis and paired-samples t-tests of the CBCL for the T1 subgroups and complete sample

Groups	CBCL total							CBCL internalizing							CBCL externalizing						
	T1	T2				95%		T1	T2				*95%*		T1	T2				*95%*	
	*M (SD)*		*t*	*df*	*p*	*CI*	*d*	*M (SD)*		*t*	*df*	*p*	*CI*	*d*	*M (SD)*		*t*	*df*	*p*	*CI*	*d*
Complete Sample	65.02 (9.58)	59.27 (10.87)	7.96	127	.001***	[4.32; 7.18]	.70	63.59 (10.38)	58.16 (11.00)	6.40	127	.001***	[3.75; 7.11]	.57	61.95 (10.29)	57.27 (10.23)	6.89	127	.001***	[3.43; 6.03]	0.61
Group 1	52.10 (5.65)	48.23 (8.11)	2.94	29	.036*	[1.17; 6.65]	.54	50.10 (5.45)	48.80 (8.47)	1.11	29	> .999	[-1.44; 4.84]	.20	51.50 (7.20)	48.47 (7.62)	2.70	29	.072	[.734; 5.33]	0.50
Group 2	70.79 (4.01)	63.28 (8.32)	6.71	38	.012*	[5.25; 9.78]	1.07	65.46 (7.97)	59.36 (9.76)	4.52	38	.012*	[3.37; 8.83]	.72	69.49 (4.44)	61.36 (8.50)	7.11	38	.012*	[5.81; 10.44]	0.71
Group 3	77.94 (4.25)	69.63 (12.05)	3.09	16	> .999	[2.88; 15.74]	.77	76.63 (5.20)	66.50 (10.13)	3.15	16	> .999	[3.28; 16.97]	.79	74.75 (5.54)	66.81 (11.45)	3.31	16	.156	[2.83; 13.04]	0.82
Group 4	62.41 (3.80)	58.22 (6.85)	3.36	31	.012*	[1.65; 6.73]	.60	64.31 (4.35)	58.53 (7.11)	4.03	31	.012*	[2.86; 8.71]	.71	58.09 (6.73)	56.13 (8.06)	1.57	31	.756	[-.583; 4.52]	0.28
Group 5	68.55 (2.66)	64.55 (8.99)	1.44	10	> .999	[-2.21; 10.21]	.43	72.73 (4.08)	67.36 (12.68)	1.38	10	> .999	[-3.28; 14.01]	.42	56.36 (6.23)	56.18 (6.35)	0.10	10	> .999	[-3.98; 4.34]	0.03

*CBCL* Child Behavior Checklist. *M* Mean, *SD* Standard deviation, *CI* Confidence Interval, *d* Effect size Cohen’s *d*. * *p* < .05, *** *p* < .001

Group 1 = Children with normative emotions and behavior; Group 2 = Children with externalizing symptoms; Group 3 = Children with multiple symptoms; Group 4 = Children with anxious-avoidant symptoms; Group 5 = Children with internalizing symptoms

**Table 3:** Descriptive analysis and paired-samples t-tests of the CNI for the T1 subgroups and complete sample

Groups	CNI						
	T1	T2					
	*M (SD)*		*t*	*df*	*p*	*CI*	*d*
Complete Sample	41.27 (19.68)	25.99 (19.46)	9.08	145	.001**	[11.95; 18.60]	0.75
Group 1	44.39 (17.36)	28.90 (20.75)	4.60	40	.012*	[8.68; 22.92]	0.72
Group 2	41.70 (21.51)	25.23 (20.60)	5.54	43	.012*	[10.48; 22.47]	0.84
Group 3	38.95 (16.21)	30.53 (19.92)	1.94	15	.408	[-.71; 17.55]	0.45
Group 4	43.18 (19.99)	25.30 (15.91)	4.86	32	.012*	[10.38; 25.38]	0.85
Group 5	22.78 (19.22)	9.44 (10.74)	2.22	8	.348	[-.52; 27.19]	0.74

*CNI* Child Neglect Index. *M* Mean, *SD* Standard deviation, *CI* Confidence Interval, *d* Effect size Cohen’s *d*. * *p* < .05, ** *p* < .001

Group 1 = Children with normative emotions and behavior; Group 2 = Children with externalizing symptoms; Group 3 = Children with multiple symptoms; Group 4 = Children with anxious-avoidant symptoms; Group 5 = Children with internalizing symptoms

### Changes in Emotional and Behavioral Problems and Severity of Child Neglect in the Subgroups

To determine the changes in emotional and behavioral problems and child neglect between T1 (pre-treatment) and T2 (post-treatment) within each subgroup, *t*-tests were performed for the individual subgroups. Tables 2 and 3 present the results for the CBCL total scores and CBCL internalizing and externalizing problems and the CNI, respectively.

**Fig. 1:**
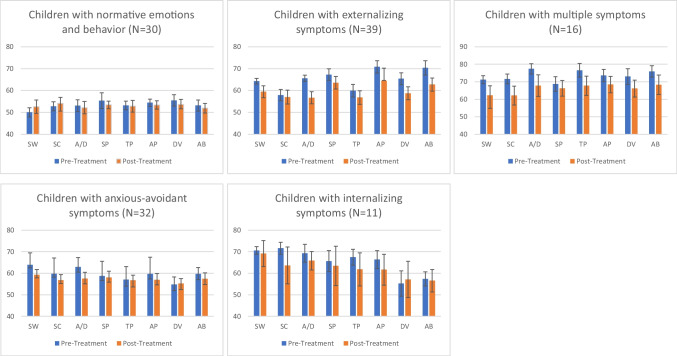
Changes in the CBCL subscale scores within the subgroups (CBCL profiles at T1) with paired samples *t*-tests at T1 and T2 (MST-CAN pre- and post-treatment). Significant changes observed for children with externalizing symptoms (SW: *t* = 3.25, *p* = .012*, A/D: *t* = 4.25, *p* = .012*, SP: *t* = 2.99, *p* = .024*, AP: *t* = 5.48, *p* = .012*, DB: *t* = 5.52, *p* = .012*, AB: *t* = 6.26, *p* = .012*); Children with multiple symptoms (SW: *t* = 3.56, *p* = .012*, SC: *t* = 3.37, *p* = .024*, A/D: *t* = 3.47, *p* = .024*, TP: *t* = 3.34, *p* = .024*, DB: *t* = 3.45, *p* = .024*, AB: *t*t = 3.17, *p* = .036*); Children with anxious-avoidant symptoms (SW: *t* = 3.05, *p* = .024*, A/D: *t* = 3.58, *p* = .012*). Note. CBCL = Child Behavior Checklist. SW = Social Withdrawal, SC = Somatic Complaints, A/D = Anxiety/Depression, SP = Social Problems, TP = Thought Problems, AP = Attention Problems, DB = Delinquent Behavior, AB = Aggressive Behavior. * *p* < .05

Figure 1 illustrates the mean changes for all the CBCL subscales per group between T1 and T2. The results for the descriptive statistics and *t*-tests for each subscale and subgroup are listed in Appendices B and C, respectively.

#### Group 1: Children with Normative Emotions and Behavior

In this group (n = 30), characterized by non-clinical scores at admission, the CBCL total scores decreased significantly from T1 to T2 with a medium effect size (*t* = 2.94, *p* = 0.036, *d* = 0.54) (Table 2). Furthermore, significant changes with a medium effect size were also observed in the CNI scores between T1 and T2 (*t* = 4.60, *p* = 0.012, *d* = 0.72) (Table 3).

#### Group 2: Children with Externalizing Symptoms

This group (n = 39) had elevated clinical scores for externalizing problems at admission. They benefited the most and demonstrated significant changes in the CBCL total score with a large effect size (*t* = 6.71, *p* = 0.012, *d* = 1.07) as well as the subscales for internalizing with a medium effect size (*t* = 4.52, *p* = 0.012, *d* = 0.72) and externalizing problems with medium effect size (*t* = 7.11, *p* = 0.012, *d* = 0.71) (Table 2). The CNI scores also changed significantly with a large effect size (*t* = 5.54, *p* = 0.012, *d* = 0.84) (Table 3), which indicated the beneficial effects for MST-CAN. Furthermore, children’s scores decreased significantly over time for the CBCL subscales of “social withdrawal” (*t* = 3.25, *p* = 0.012, *d* = 0.52), “anxiety/depression” (*t* = 4.25, *p* = 0.012, *d* = 0.68), “social problems” (*t* = 2.99, *p* = 0.024, *d* = 0.48), “attention problems” (*t* = 5.48, *p* = 0.012, *d* = 0.88)), “delinquent behavior” (*t* = 5.48, *p* = 0.012, *d* = 0.88), and “aggressive behavior” (*t* = 6.26, *p* = 0.012, *d* = 1.00) (Fig. 1, Appendices B and C). Effect sizes ranged from medium to large.

#### Group 3: Children with Multiple Symptoms

This group (n = 17) reported high scores in a clinical range on all the subscales and met the criteria for the CBCL dysregulation profile, which indicated severe psychopathology (Deutz et al., 2020; Dölitzsch et al., 2016). Beneficial changes were observed at a CBCL subscale level, although not for the CBCL total scores and CNI (Tables 2 and 3). At the CBCL subscale level, scores decreased significantly over time with medium to large effect sizes for “social withdrawal” (*t* = 3.56, *p* = 0.012, *d* = 0.89), “somatic complaints” (*t* = 3.37, *p* = 0.024, *d* = 0.84), “anxiety/depression” (*t* = 3.47, *p* = 0.024, *d* = 0.87), “thought problems” (*t* = 3.34, *p* = 0.024, *d* = 0.83), “delinquent behavior” (*t* = 3.45, *p* = 0.024, *d* = 0.86), and “aggressive behavior” (*t* = 3.17, *p* = 0.036, *d* = 0.79) (Fig. 1, Appendices B and C).

#### Group 4: Children with Anxious-Avoidant Symptoms

This group (n = 32) was characterized by scores in a clinical range on the scales for “social withdrawal” and “anxiety/depression.” Significant changes with medium effect sizes were observed over time for the CBCL total score (*t* = 3.36, *p* = 0.012, *d* = 0.60) and internalizing problems (*t* = 4.03, *p* = 0.012, *d* = 0.71) (Table 2). Furthermore, the CNI score also changed significantly over time with a large effect size (*t* = 4.86, *p* = 0.012, *d* = 0.85) (Table 3). At a subscale level, scores decreased significantly over time with medium effect sizes for “social withdrawal” (*t* = 3.05, *p* = 0.024, *d* = 0.54) and “anxiety/depression” (*t* = 3.58, *p* = 0.012, *d* = 0.63) (Fig. 1, Appendices B and C).

#### Group 5: Children with Internalizing Symptoms

Children in this group (n = 11) primarily exhibited internalizing problems with high scores in the clinical range for the subscales assigned to internalizing problems. No significant changes were observed over time in the CBCL, total score, or subscale scores (Table 2, Fig. 1, Appendices B and C). Additionally, no significant changes were observed in the CNI (Table 3).

### Subgroup Changes Between T1 and T2

To determine the changes across the subgroups, clusters were identified at T2 (LICUR steps 1 and 2). Subsequently, the clusters were compared with those identified at T1 (structural change), and their developmental paths between the two measurement points were analyzed (individual change) (LICUR step 3). Finally, the clusters identified at T2 were examined to distinguish their characteristics in comparison to T1.

#### Identification of Cluster at T2

**Fig. 2:**
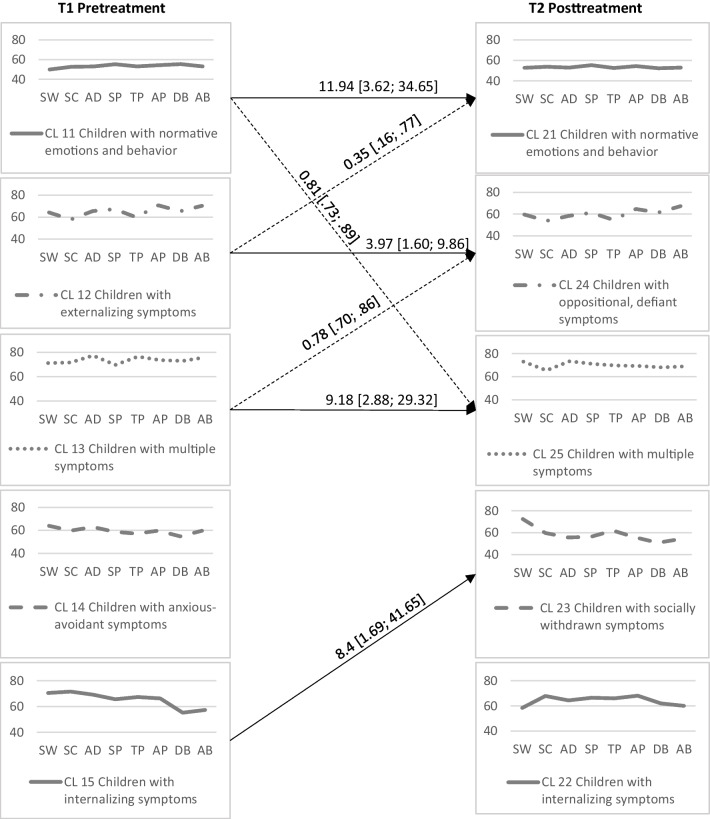
Cluster centroids at T1 and T2 and the transitions. *Note*. Operating factors: SW = Social Withdrawal, SC = Somatic Complaints, AD = Anxiety/Depression, SP = Social Problems, TP = Thought Problems, AP = Attention Problems, DB = Delinquent Behavior, AB = Aggressive Behavior. → Significantly more transitions ⇢ Significantly less transitions. The numbers next to the arrows represent the odds ratios (ORs) and 95% CIs (significantly more transitions: OR > 1.0; significantly less transitions: OR < 1.0)

We performed a cluster analysis at T2. No multivariate outliers were found as assessed by the Mahalanobis distance (*p* > 0.001). The five-cluster solution (see Fig. 2 and detailed results in Appendix D) was chosen owing to its content aspects and considering that it met Bergman et al.’s criteria (2003).

##### Group 1: Children with Normative Emotions and Behavior

The first and largest group (n = 62) comprised children with low scores in the normal range across all subscales. Scores indicated that these children did not have psychopathological symptoms at the end of the MST-CAN treatment.

##### Group 2: Children with Internalizing Problems

Children in the second group (n = 14) scored in the clinical range on the subscales “somatic complaints,” “anxiety/depression,” “social problems,” and “thought problems.” These findings indicated a group with predominantly internalizing problems.

##### Group 3: Children with Socially Withdrawn Symptoms

The third group was the smallest (n = 8). Children scored high on the subscale “social withdrawal” and in the borderline clinical range on the subscales “anxiety/depression” and “thought problems.” All other scores were within the normal ranges. These results indicated that the children were socially isolated and withdrawn.

##### Group 4: Children with Oppositional, Defiant Symptoms

The fourth group (n = 25) comprised children with scores in a clinical range for the subscales “attention problems” and “aggressive behavior.” Additionally, scores were in a borderline clinical range for the subscales “social problems” and “delinquent behavior.” All other scores were within the normal ranges. These findings revealed a clinical picture of children who were oppositional and defiant.

##### Group 5: Children with Multiple Symptoms

The fifth group (n = 19) scored high in the clinical range on all the subscales. The T-scores for the subscales of “anxiety/depression,” “attention problems,” and “aggressive behavior” were above 67. Therefore, children met the criteria for the dysregulation profile, which indicated that they still exhibited severe psychopathology at the end of treatment, despite significant reductions in “somatic complaints,” “anxiety/depression,” “thought problems,” “delinquent behavior,” and “aggressive behavior.” Detailed results regarding the subgroup profiles at T2 (means and standard deviations for the subscales) are presented in Appendix D.

#### Structural Change

Figure 2 illustrates the two cluster solutions for T1 (pre-treatment) and T2 (post-treatment). We identified five similar patterns. Clusters were arranged horizontally based on similarity. High structural stability was observed between the clusters across the measurement points, measured by the distances between the corresponding clusters (0.00 < SS < 0.07).

#### Individual Change

Figure 2 also illustrates the individual developmental paths between the clusters and the two measurement points. We identified three stable developmental types (transitions occurring with a high probability; solid arrows in Fig. 2) for the clusters CL11 (children with normative emotions and behavior), CL12 (externalizing symptoms), and CL13 (multiple symptoms) in their respective corresponding clusters at the second measurement point. These findings indicated individual stability in the profiles for these groups. An additional developmental type that did not run between the two corresponding clusters was found, which indicated a profile change. This was between clusters CL15 (children with internalizing symptoms) and CL23 (children with socially withdrawn symptoms). Developmental antitypes (high probability of no transition occurring; dashed arrows in Fig. 2) were found between dissimilar clusters, namely between CL11 (children with normative emotions and behavior) and CL25 (children with multiple symptoms), CL12 (children with externalizing symptoms) and CL21 (children with normative emotions and behavior), and CL13 (children with multiple symptoms) and CL24 (children with oppositional, defiant symptoms). These antitypes were used as confirmation of the above-mentioned individual profile stability for children with normative emotions and behavior, externalizing problems, and multiple systems between T1 and T2. These antitypes indicated the unlikeliness of a profile change.

#### Subgroup Characteristics (T2 Subgroups)

Investigation of the subgroup characteristics did not reveal any significant differences in demographic variables. Detailed results of the group comparisons are provided in Appendices E and F.

## Discussion

This study aimed to investigate the changes in emotional and behavioral problems, severity of child neglect, and subgroup changes among children and adolescents treated with MST-CAN. It utilized a dual approach that combined both variable-centered and person-centered methods. These findings provide novel evidence that children and adolescents differ in their responses to MST-CAN and show distinct subgroup changes. Of the five subgroups, four benefited from treatment in at least two outcome measures and showed differential changes in emotional and behavioral problems. Furthermore, three subgroups also showed reductions in child neglect, highlighting that most families benefited from treatment. The subgroups identified at the beginning of the treatment reappeared at the end, albeit with sharper symptoms. This may be a result of changes in various symptom scales due to the therapy. Additionally, three subgroups exhibited high individual stability, indicating the stability of symptom classes over time, while one group transitioned into another subgroup. The findings for each of these five subgroups are discussed below. Overall, the results point to the benefit of MST-CAN for a wide range of children and adolescents with various psychopathologies. Due to their varying psychopathologies, we can assume that they exhibit different clinical needs, which lead to different treatment responses. The results of differential treatment responses to family-based interventions were consistent with those of prior research, suggesting differential treatment responses among children and adolescents in high-risk families. Keles et al. (2021) found heterogeneous trajectories among adolescents with serious and persistent antisocial behaviors treated with MST Standard in Norway. Mertens et al. (2017) discovered different treatment trajectories in adolescents with externalizing behaviors. Outside the MST context, divergent treatment trajectories were observed among adolescents with severe behavioral and mental health problems assigned to an attachment-based and trauma-informed parent program (Pasalich et al., 2022).

The MST-CAN was most beneficial for children with externalizing symptoms. Changes were observed in all scales with medium to large effects, including child neglect, with the exception of two that measured emotional and behavioral problems. This result corresponds with those of Pasalich et al. (2022), who reported that adolescents with severe externalizing problems showed the fastest and largest improvement in treatment. The favorable outcomes in children with externalizing symptoms in the present study could be attributed to the origin of the MST Standard, which was primarily designed to treat adolescents with externalizing problems. Notably, Zhang and Slesnick (2018) concluded that family systems therapy is especially effective in reducing externalizing problem behavior when compared with a non-family focused control treatment. The findings might provide initial indications that outreach and/or systemic approaches are particularly effective for children and adolescents with externalizing symptoms (Boege et al., 2015; Zhang & Slesnick, 2018). In general, MST programs have a relatively strong focus on parenting strategies and adult behavior. Therefore, caregivers may establish more rules in the home and monitor their children more heavily, while at the same time show more warmth and nurturing behavior towards their children. The move towards an authoritative parenting style could therefore be a mechanism for changing the children’s externalizing symptoms. Further, it is possible that the results are related to the ages of the children and adolescents in this study (age range: 6–17 years; mean age: 10.3 years). Specifically, adults may tend to focus on externalizing symptoms or fail to recognize internalizing symptoms in 6- to 17-year-olds. Accordingly, the adult caregivers who conducted the assessments and the school employees who played an important role in the referral procedure may have more readily recognized externalizing symptoms in the participants than internalizing ones.

Furthermore, children with externalizing symptoms had a high likelihood of remaining in the equivalent subgroup at the end of the treatment, indicating that they likely showed oppositional, defiant symptoms as the treatment finished. Zhang and Slesnick (2018) reported that adolescents transitioned from the externalizing class to the normative class 18 months after treatment. While these two different timelines cannot be compared, it would be interesting to know whether children treated with MST-CAN may stop showing clinically significant symptoms after a longer period of time.

For children with multiple symptoms, the MST-CAN was beneficial for various emotional and behavioral subscales with medium to large effects observed among the changes; however, this was not the case for child neglect and overall scales, indicating some symptom improvements. A parallel can be drawn from Pasalich et al.’s study (2022), which found a moderate treatment response with gradual improvements in a group of children with co-occurring externalizing and internalizing problems. Within the context of Weisz et al.’s meta-analysis (2017), our results seem to affirm the benefits of MST-CAN for children with severe psychopathology. In the meta-analysis, only small effects and no significant differences from zero were observed in children and adolescents with multiple symptoms. This may be due to the nature of the MST-CAN’s complex treatment program, which is specifically designed to treat families with multiple needs.

Further analyses of the subgroup changes revealed stability over time. This indicates that the children in this group still show signs of severe psychopathology even after treatment. This finding is consistent with Halliday-Boykins et al.’s study (2004), in which youths with severe psychopathology were at risk of maintaining their symptoms at a high level and benefiting less from treatment. The results point to a particularly vulnerable subgroup of children. Children in this group already displayed a high comorbidity at the beginning of the treatment, which might make it more difficult to induce changes. In a previous study on MST-CAN (Buderer et al., 2024), multiple symptoms in children were associated with higher mental health problems in parents. It is possible that parents in this group, due to their own burdens, may implement interventions less effectively and swiftly, which is also why more significant changes were not possible within a short period of time. Even though these results seem plausible, particularly in light of corresponding studies, it must be noted that the small sample size of N = 16 for this group limits the interpretability of the findings.

Children with anxious-avoidant symptoms benefited in terms of their specific symptoms with medium effects and child neglect with a large effect. On the other scales they exhibited values in a normal range leading presumably to non-significant changes over time. Research to draw parallels from studies with similar sample and group characteristics is lacking. Weisz et al. (2017) found the highest effects of psychological treatment for children and adolescents with anxiety. However, our findings are inconsistent with this perspective, which might be owing to the more complex nature of a high-risk sample.

Treatment was still beneficial in improving overall emotional and behavioral problems and reducing child neglect for children with normative emotions and behaviors. We attribute the existence of a group without psychopathological findings to the fact that children can remain resilient despite experiencing maltreatment or may develop symptoms only later in life (Fonagy et al., 2014). To the best of our knowledge, no previous research has contextualized these change values. Halliday-Boykins et al. (2004) found a subclinical group of youths following a psychiatric crisis; however, they were unable to further statistically analyze it due to the small group size. This normative group showed individual subgroup stability, which may be evident and suggests that these children continue to remain resilient within the context of child abuse and neglect.

For children with internalizing symptoms, no significant changes were found over time for the measured outcomes. This might indicate a non-responder group, but it could also be due to non-clinical values at the beginning of the treatment on some scales. We did not find supportive evidence that family-based therapy was not beneficial for children with internalizing symptoms in comparable samples. However, this result must be considered in light of the small subgroup size (N = 11), which may bias the representativeness of the outcomes. Therefore, interpretation must be made with caution.

Our second analysis revealed subgroup changes. Children with internalizing symptoms showed an increased likelihood of belonging to the subgroup of children with socially withdrawn symptoms after treatment. This result may suggest that social withdrawal as a specific symptom could not be adequately addressed during treatment or that these children and adolescents exhibit specific temperamental traits that make it difficult to reach them. However, there are few studies with which to compare this result.

Our results regarding the differential treatment responses for reducing child neglect are particularly noteworthy. To the best of our knowledge, this is the first study to examine this. These results could provide an initial indication that children may benefit in different ways from a treatment program for maltreatment depending on their psychopathological symptoms. Further studies should examine the relationships between various psychopathologies and the effects of treatment in children.

This study has several limitations. First, the study was not designed as an RCT, which restricts the generalizability of the results. Ultimately, we cannot conclude that the changes in children’s psychopathology and neglect are attributable to the intervention program. However, with MST-CAN as a standardized treatment program executed in a natural setting with real treatment conditions, the clinical representativeness and significance of the results is enhanced (Weisz et al., 2005). Although Switzerland has some legal peculiarities, these results can be generalized to other Western countries with similar ethnic and racial groups. To ensure that MST-CAN is also beneficial for other groups, the results must be replicated in samples that include children and families from underrepresented backgrounds.

Second, we assessed children’s psychopathology through parent reports via questionnaires. We did not include the children’s perspectives in the assessment, which could differ from their parent’s perspectives. Therefore, we cannot exclude the possibility that the changing values are due to a shift in how parents perceive their children. As they address their traumatic experiences during treatment, they may become more empathetic towards their children and see them as less difficult or oppositional (e.g. in children with externalizing symptoms).

Previous studies (Buderer et al., 2024; Hefti et al., 2020) reported that a significant proportion of parents of families referred to the MST-CAN suffered from mental health problems, which could bias their reports regarding children’s psychopathology (De Los Reyes & Kazdin, 2005). Furthermore, in a sensitive context, such as child protection, parents may be inclined to present themselves in a more favorable light (van de Mortel, 2008). Nevertheless, we followed a multi-informant approach (De Los Reyes et al., 2015) and engaged with an external professional caseworker involved with the families to assess child neglect and the existence and typology of child maltreatment. This was a strength of the study. However, future studies should consider additional assessments and methods. Further, this study’s data may also have been biased by the fact that the 24 therapists treated a wide variation of cases. We did not account for therapist variation in the study, although it might have impacted treatment outcomes.

Third, cluster analysis has some limitations. A significant limitation is the false-positive identification of clusters and the tendency to discover two clusters in a dataset (Tokuda et al., 2022). Therefore, the validation and replication of the identified 5-cluster solutions in datasets from other studies are essential. Some of the subgroups were smaller than the 20 cases, which limits the power of the cluster analysis. Furthermore, the cluster solution must be interpreted with caution. However, the Ward method was the only suitable method considering the exploratory nature of this study and its sample size (Bacher et al., 2010). By applying the LICUR procedure as a more advanced method, a more sophisticated validation of clusters was possible, which is a strength of this study.

## Clinical Implications

The study’s findings provide valuable insights for clinical practice and can assist clinicians in tailoring interventions for children’s individual needs. Results on differential treatment responses emphasize the benefits of MST-CAN for children and adolescents with externalizing symptoms. MST-CAN primarily focuses on adult behavior in the treatment model. Interventions focused on parents include parenting skills (e.g., positive parenting strategies, establishing rules, and increasing the monitoring of their children), taking responsibility and apologizing to the family for the maltreatment of the children, and creating and maintaining safety plans. Additionally, parents may participate in individual treatment to resolve their own traumas, decrease substance misuse, and learn skills to manage anger and implement family-based problem solving and communication. These treatment strategies and changes in parental behavior, combined with the involvement of the natural ecology in social support, may be particularly helpful for children and adolescents in reducing externalizing symptoms. Furthermore, these results shed light on a particularly vulnerable subgroup of children and adolescents with multiple symptoms. These individuals should be identified early during treatment and may require additional care and intervention even after MST-CAN treatment. For these children and families, limiting the consequences of mental illness and collaborating with other support systems to provide the necessary assistance to ensure societal participation is crucial. Children and adolescents with internalizing symptoms may be at risk of not benefiting from treatment and exhibiting pronounced symptoms of social withdrawal at the end of treatment. To address their needs effectively, MST-CAN treatments to improve the parent–child relationship, additional social skills training with skill-building exercises (de Mooij et al., 2020), or youth-focused treatment may be helpful. Children without psychopathological symptoms also benefit from MST-CAN, which underscores the significance of treatment regarding the overarching goal of preventing and reducing child maltreatment. Parent and family changes may have a preventative function regarding mental health difficulties. In high-risk families where children experience mental health difficulties, focusing on the consequences and effects of child maltreatment and promoting resources may be helpful. Furthermore, this may serve as prevention for the recurrence of maltreatment. In outlining the clinical implications of this study, it is important to note that generalizing the findings to other treatment programs will require the psychopathology profiles to be replicated within those specific programs.

## Conclusion

Our results highlight the benefits of MST-CAN for children and adolescents with differing mental health problems. These findings further support evidence that children and adolescents differ in their response to multisystemic treatment within a high-risk context. Hence, future studies should incorporate person-centered methods in their analyses. In particular, for differential responses regarding the reduction of child neglect, this study may serve as a preliminary template and stimulate further research. Children who benefit less should be identified early during treatment and treated with additional interventions. For children with internalizing symptoms, social skills training might be beneficial to prevent them from developing socially withdrawn symptoms. Children with multiple symptoms may benefit from additional support, even after treatment, to reduce the long-term effects of severe psychopathology and limit societal participation. Our results contribute to a better understanding of the clinical needs of children and adolescents from families treated with MST-CAN. These results may also be of interest to other manualized treatment programs that target children and adolescents in a high-risk context, justifying standardized treatment programs.

## Supplementary Information

Below is the link to the electronic supplementary material.

Supplementary file1 (DOCX 40.3 KB)
