# Supplementary material for: Differential Treatment Responses of Maltreated and Neglected Children and Adolescents Following an Evidence-based Multisystemic Intervention
Source: Res Child Adolesc Psychopathol. 2024 Oct 14;53(1):69–84. doi: 10.1007/s10802-024-01248-z (PMC11761468; doi:10.1007/s10802-024-01248-z)
Supplement: Supplementary file 1 — Supplementary file1 (DOCX 40.3 KB) [file 10802_2024_1248_MOESM1_ESM.docx]

**Appendix A**Flowchart depicting participation and analysis

Enrollment in MST-CAN

July 2011 to November 2023

*n* = 313

Agreed to participate in the study

T1: *n* = 214

T2: *n* = 181

Discharge from MST-CAN

Lack of engagement

*n* = 15

Out-of-home placement

*n* = 5

Administrative withdrawals

*n* = 4

Lack of funding

*n* = 3

Family relocation

*n* = 2

Research Question 1

CBCL T1: *n* = 208
*t*-tests T1 – T2 : *n* = 128
CNI *t*-tests T1 – T2: *n* = 146

Research Questions 2 and 3

CBCL T2: *n* = 128

**Appendix B**
Descriptive analysis of the Child Behavior Checklist subscales for the subgroups (T1 profiles).

| Measure | Group 1 | | Group 2 | | Group 3 | | Group 4 | | Group 5 | |
| --- | --- | --- | --- | --- | --- | --- | --- | --- | --- | --- |
|  | T1 | T2 | T1 | T2 | T1 | T2 | T1 | T2 | T1 | T2 |
|  | *M (SD)* | | *M (SD)* | | *M (SD)* | | *M (SD)* | | *M (SD)* | |
| Social Withdrawal | 50.00 (9.60) | 52.60 (4.60) | 64.26 (8.09) | 59.44 (8.77) | 71.19  (7.85) | 62.25  (8.33) | 63.91 (5.99) | 59.25 (8.13) | 70.55  (7.31) | 69.18  (13.60) |
| Somatic Complaints | 52.77 (5.31) | 54.13 (7.21) | 57.87 (8.90) | 56.97 (9.49) | 71.63  (7.35) | 62.13  (10.26) | 59.75 (8.44) | 56.78 (6.79) | 71.64  (5.52) | 63.64  (11.47) |
| Anxiety/ Depression | 53.03 (4.37) | 52.17 (5.05) | 65.56 (8.64) | 59.69 (8.54) | 77.38 (6.60) | 67.81 (8.79) | 62.91 (6.51) | 57.50 (8.11) | 69.27 (6.18) | 65.82 (10.61) |
| Social Problems | 55.37 (7.00) | 53.43 (5.22) | 67.26 (8.44) | 63.54 (9.05) | 68.69 (8.35) | 66.25  (9.07) | 58.72 (7.78) | 58.03 (6.23) | 65.64 (8.78) | 63.45  (12.52) |
| Thought Problems | 53.17 (6.85) | 52.83 (5.82) | 59.92 (10.09) | 56.77 (7.25) | 76.5  (6.13) | 67.75  (10.46) | 57.06 (7.23) | 56.69 (7.77) | 67.45  (4.87) | 61.82  (7.77) |
| Attention Problems | 54.43 (5.90) | 53.40 (5.06) | 70.82 (6.44) | 64.44 (7.25) | 73.56 (9.37) | 68.38 (9.75) | 59.69 (6.42) | 56.97 (7.04) | 66.36 (5.48) | 61.64 (7.05) |
| Delinquent Behavior | 55.47 (5.39) | 53.57 (5.60) | 65.38 (6.60) | 58.72 (7.50) | 73.00 (7.52) | 66.13 (11.42) | 54.75 (5.59) | 55.25 (5.93) | 55.27 (6.71) | 57.09 (7.30) |
| Aggressive Behavior | 53.13 (4.08) | 51.90 (2.91) | 70.36 (6.56) | 62.72 (8.86) | 75.88 (7.15) | 68.25 (9.78) | 59.66 (6.71) | 57.41 (8.47) | 57.36 (5.80) | 56.55 (5.12) |

*Note.* General scores for the Child Behavior Checklist scales are normal for T < 60, borderline clinical range for T
between 60 and 63, clinical for T > 64. *M* = Mean, *SD* = Standard deviation.
Group 1 = Children with normative emotions and behavior, Group 2 = Children with externalizing symptoms, Group 3 = Children with multiple symptoms, Group 4 = Children with anxious-avoidant symptoms, Group 5 = Children with internalizing symptoms.

**Appendix C**
Paired-sample t-tests of the Child Behavior Checklist subscales at T1 and T2 (pre- and post-treatment) for the subgroups (T1 profiles).

| Measure | Group 1 | | | Group 2 | | | Group 3 | | | Group 4 | | | Group 5 | | |
| --- | --- | --- | --- | --- | --- | --- | --- | --- | --- | --- | --- | --- | --- | --- | --- |
|  | *t* | *df* | *p* | *t* | *df* | *p* | *t* | *df* | *p* | *t* | *df* | *p* | *t* | *df* | *p* |
| Social Withdrawal | -1.195 | 29 | >.999 | 3.254 | 38 | .012* | 3.564 | 15 | .012* | 3.049 | 31 | .024* | 0.313 | 10 | >.999 |
| Somatic Complaints | -1.072 | 29 | >.999 | 0.579 | 38 | >.999 | 3.371 | 15 | .024* | 1.880 | 31 | .42 | 2.178 | 10 | .324 |
| Anxiety/ Depression | 0.765 | 29 | >.999 | 4.253 | 38 | .012* | 3.473 | 15 | .024* | 3.580 | 31 | .012* | 0.993 | 10 | >.999 |
| Social Problems | 1.373 | 29 | >.999 | 2.992 | 38 | .024* | 1.047 | 15 | >.999 | 0.647 | 31 | >.999 | 0.602 | 10 | >.999 |
| Thought Problems | 0.282 | 29 | >.999 | 1.813 | 38 | .468 | 3.336 | 15 | .024* | 0.273 | 31 | >.999 | 2.946 | 10 | .084 |
| Attention Problems | 1.080 | 29 | >.999 | 5.475 | 38 | .012* | 2.746 | 15 | .096 | 2.588 | 31 | .084 | 1.755 | 10 | .66 |
| Delinquent Behavior | 1.460 | 29 | .924 | 5.516 | 38 | .012* | 3.445 | 15 | .024* | -.420 | 31 | >.999 | -0.731 | 10 | >.999 |
| Aggressive Behavior | 1.963 | 29 | .36 | 6.261 | 38 | .012* | 3.173 | 15 | .036* | 1.760 | 31 | .528 | 0.477 | 10 | >.999 |

*Note.* * *p* < .05.
Group 1 = Children with normative emotions and behavior, Group 2 = Children with externalizing symptoms, Group 3 = Children with multiple symptoms, Group 4 = Children with anxious-avoidant symptoms, Group 5 = Children with internalizing symptoms.

**Appendix D**
Between group differences: Means and standard deviations of characteristics
across the five subgroups (T2 profiles).

| Measure | Group 1: *n* = 19  *M (SD)* | Group 2: *n* = 14  *M (SD)* | Group 3: *n* = 62  *M (SD)* | Group 4: *n* = 8  *M (SD)* | Group 5: *n* = 25  *M (SD)* | *F* | *p* |
| --- | --- | --- | --- | --- | --- | --- | --- |
| Social Withdrawal | 73.00 (7.96) | 58.43 (5.95) | 52.79 (3.49) | 72.50 (7.80) | 59.64 (5.26) | 67.95 | <.001 |
| Somatic Complaints | 65.47 (11.07) | 67.93 (8.78) | 53.85 (5.96) | 59.75 (9.44) | 53.80 (4.27) | 18.81 | <.001 |
| Anxiety/  Depression | 73.37 (5.61) | 64.43 (7.59) | 53.00 (4.71) | 62.63 (5.48) | 58.36 (7.95) | 46.77 | <.001 |
| Social Problems | 71.11 (10.14) | 66.50 (6.49) | 55.39 (6.09) | 56.13 (4.88) | 61.24 (7.43) | 22.04 | <.001 |
| Thought Problems | 69.74 (8.52) | 66.07 (9.89) | 52.66 (5.44) | 62.00 (4.14) | 54.64 (5.79) | 33.00 | <.001 |
| Attention Problems | 69.26 (9.13) | 68.14 (3.57) | 54.47 (5.20) | 55.75 (5.20) | 64.68 (6.65) | 34.61 | <.001 |
| Delinquent Behavior | 68.11 (8.74) | 62.00 (6.68) | 52.37 (3.21) | 50.88 (2.48) | 61.40 (6.75) | 39.56 | <.001 |
| Aggressive Behavior | 69.00 (9.26) | 60.07 (5.77) | 52.97 (4.06) | 54.25 (4.86) | 67.36 (7.82) | 40.67 | <.001 |

*Note.* General scores for the Child Behavior Checklist scales are normal for T < 60, borderline clinical range for T between 60 and 63, clinical for T > 64. *M* = Mean, *SD* = Standard deviation.
Group 1 = Children with multiple symptoms, Group 2 = Children with internalizing symptoms, Group 3 = Children with normative emotions and behavior, Group 4 = Children with socially withdrawn symptoms, Group 5 = Children with oppositional-defiant symptoms.

**Appendix E**
Chi-squared tests on pretreatment differences in characteristics across the five subgroups (T2 profiles).

| Characteristics | Group 1:  *n* (%) | Group 2:  *n* (%) | Group 3:  *n* (%) | Group 4:  *n* (%) | Group 5:  *n* (%) | *X*² [95% CI] | |
| --- | --- | --- | --- | --- | --- | --- | --- |
| Female | 30 (48.4%) | 5 (35.7%) | 3 (37.5%) | 10 (40.0%) | 10  (52.6%) | 1.65 [.809,.824] | |
| Migration background | 21 (33.9%) | 4  (28.6%) | 5  (62.5%) | 8  (32.0%) | 7  (36.8%) | 3.07  [.551, .570] | |
| Single parenthood | 33 (53.2%) | 5  (35.7%) | 4 (50.0%) | 10 (40.0%) | 15 (78.9%) | 8.51 [.066,.076] |  |
| Parent unemployed | 28 (45.9%) | 3  (21.4%) | 2  (25.0%) | 8  (32.0%) | 11  (57.9%) | 6.75  [.146, .160] | |
| Parent without graduation | 5  (3.1%) | 0  (0%) | 0  (0%) | 1  (4%) | 2  (10.5%) | 8.21  [.375, .394] | |
| Multiple children in the household | 26  (60.5%) | 6  (66.7%) | 4  (80.0%) | 10 (58.8%) | 9  (64.3%) | .913  [.937, .946] | |
| Referral because of child neglect | 25 (54.3%) | 7  (77.8%) | 1 (20.0%) | 9 (47.4%) | 10  (66.7%) | 5.64  [.226, .242] | |

*Note.* *M* = Mean, *SD* = Standard deviation.
Group 1 = Children with multiple symptoms, Group 2 = Children with internalizing symptoms, Group 3 = Children with normative emotions and behavior, Group 4 = Children with socially withdrawn symptoms, Group 5 = Children with oppositional-defiant symptoms.

**Appendix F**One-way analysis of variance (ANOVA) of the pre-treatment differences in age across the five subgroups (T2 profile).

| Measure | Group 1:  *M* *(SD)* | Group 2:  *M* *(SD)* | Group 3:  *M* *(SD)* | Group 4:  *M* *(SD)* | Group 5:  *M* *(SD)* | *F* | *p* |
| --- | --- | --- | --- | --- | --- | --- | --- |
| Age | 9.98 (3.36) | 9.93 (3.63) | 12.75  (2.66) | 8.86 (2.97) | 10.37 (2.93) | 2.309 | .062 |

*Note.* *M* = Mean, *SD* = Standard deviation.
Group 1 = Children with multiple symptoms, Group 2 = Children with internalizing symptoms, Group 3 = Children with normative emotions and behavior, Group 4 = Children with socially withdrawn symptoms, Group 5 = Children with oppositional-defiant symptoms.
